# Asymmetrical diacylglycerol dynamics on the cytosolic and lumenal sides of a single endomembrane in living cells

**DOI:** 10.1038/srep12960

**Published:** 2015-08-12

**Authors:** Yoshibumi Ueda, Hideo Ogiso, Moritoshi Sato, Yoshio Umezawa, Toshiro Okazaki, Toshihide Kobayashi

**Affiliations:** 1 Lipid Biology Laboratory, RIKEN, 2-1, Hirosawa, Wako-shi, Saitama 351-0198, Japan; 2 Department of Hematology and Immunology, Kanazawa Medical University, 1-1 Daigaku, Uchinada, Kahoku, Ishikawa 920-0265, Japan; 3 Department of General Systems Studies, School of Arts and Sciences, The University of Tokyo, 3-8-1 Komaba, Meguro-ku, Tokyo 153-8902, Japan; 4 Department of Chemistry, School of Science, The University of Tokyo, 7-3-1 Hongo, Bunkyo-ku, Tokyo, Japan; 5 INSERM U1060-Université Lyon1, 69621 Villeurbanne, France

## Abstract

The elucidation of lipid dynamics on the cytosolic and lumenal sides of a single endomembrane has been challenging in living cells because of the lack of appropriate methods. Diacylglycerol (DAG) is a lipid second messenger that is produced by enzymes that reside on both the cytosolic and lumenal sides of the endomembrane. In the present study, we attempted to observe both the cytosolic and lumenal DAG dynamics at endomembranes including the Golgi apparatus and the endoplasmic reticulum in Madin-Darby canine kidney (MDCK) cells. We developed a Förster resonance energy transfer (FRET)–based probe to detect DAG at the luminal side (lumenal DAG) of endomembranes. In combination with the FRET-based cytosolic DAG probe that has already been established, it was found that lumenal DAG is generated in a calcium-dependent manner by thapsigargin, which increases cytosolic calcium concentrations. In contrast, DAG production at the cytosolic side of endomembranes did not occur under the same experimental conditions. The thapsigargin-induced DAG generation was abolished by treatment with an inhibitor of sphingomyelin synthase (SMS) and phosphatidylcholine-specific phospholipase C (PC-PLC), which produce lumenal DAG. Thus, we have established a successful method for monitoring both cytosolic and lumenal DAG dynamics at the endomembrane in living cells.

Diacylglycerol (DAG) plays important roles in a variety of cellular functions, such as cell differentiation, cell growth, and vesicular trafficking^1^,^2^,^3^. Several fluorescent probes have been developed to visualize DAG dynamics in single living cells. These probes are expressed in the cytoplasm by transfection with corresponding plasmids. Fusion proteins of green fluorescent protein (GFP) and DAG-binding domains derived from protein kinase C (PKC) have been used as indicators for DAG accumulation in the cellular membrane. Translocation of the fusion proteins from the cytosol to the membrane has been explained to reflect the DAG accumulation^4^,^5^. FRET-based DAG probes have also been developed^6^,^7^,^8^. FRET probes can be targeted to desired organelles and thus give higher spatial resolution of the signal. These probes have revealed DAG dynamics in individual organelle membranes, such as the Golgi apparatus, plasma membrane, and mitochondria.

DAG is produced by phospholipase C and phosphatidate phosphatase on the cytosolic side of cellular membranes, including the plasma membrane^6^, the Golgi apparatus^8^,^9^ and mitochondria^10^. DAG is not only produced on the cytosolic side of these membranes, but it is also produced on the lumenal side of the cell membrane by other DAG-generating enzymes, such as sphingomyelin synthase^11^. Furthermore, DAG is converted to triacylglycerol by DAG acyltransferase on the lumenal side of the endoplasmic reticulum^12^. Therefore, DAG is expected to reside on both the cytosolic and the lumenal sides of the membrane.

To date, all available DAG probes only detect DAG on the cytoplasmic leaflet. In the present study, to establish a method to reveal DAG dynamics on both sides of endomembrane, we developed a novel DAG probe based on Förster resonance energy transfer (FRET) that detects lumenal DAG. Using this probe and probes to detect cytosolic DAG, we demonstrated the calcium-induced asymmetrical DAG production at endomembranes such as those of the Golgi apparatus and the endoplasmic reticulum.

## Results

**Figure 1:**
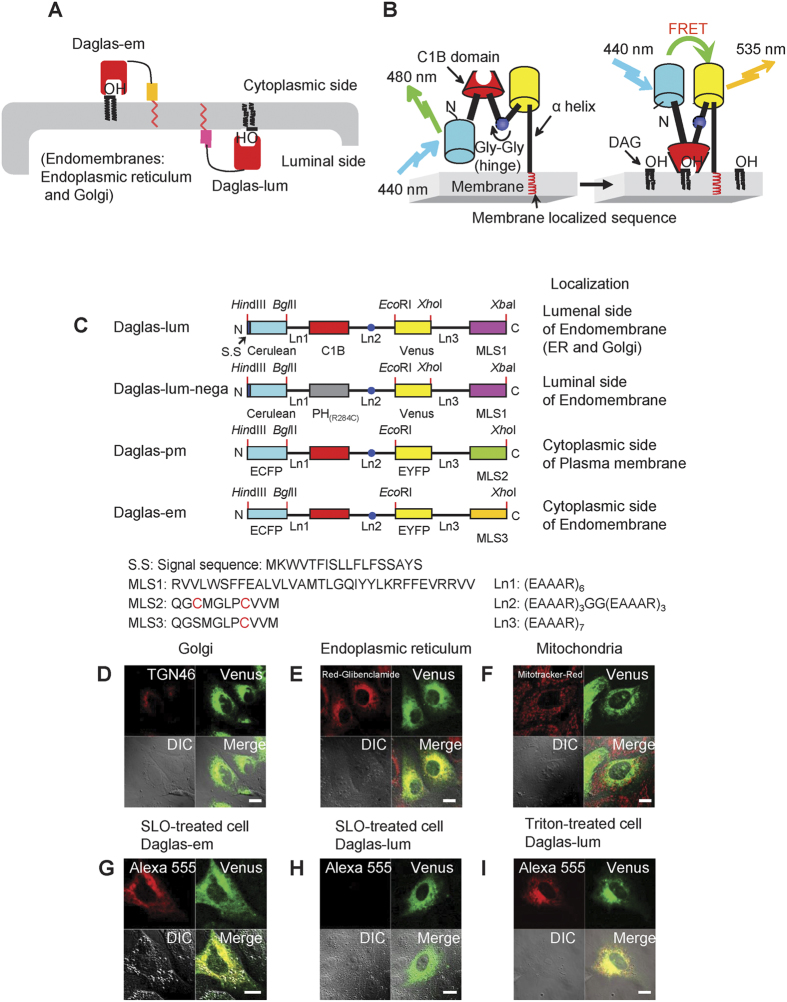
FRET-based DAG probes in living cells. (**A**) The strategy to observe DAG dynamics on the cytosolic and lumenal sides of endomembrane using FRET-based DAG probes. (**B**) Schematic representation of the detection of DAG by Daglas. Cerulean and Venus are different-colored mutants of green fluorescence protein from *Aequorea victoria* with mammalian codons and additional mutation. Upon binding of DAG to the C1B within Daglas, a filp-flop-type conformational change of Daglas takes place, resulted in the enhancement of FRET from Cerulean to Venus. (**C**) DAG binding domain is derived from rabbit protein kinase Cβ (amino acids 99-154). Ln1 and Ln3 are alpha helical rigid linkers. Ln2 has a glycine-glycine motif between EAAAR linkers. MLS1, MLS2 and MLS3 are membrane localization sequences to lumenal side of endomembrane, inner leaflet of plasma membrane, and cytosolic side of endomembranes, respectively. The amino acid sequences of the localization signals are shown at the bottom. (**D**–**F**) Subcellular localization of Daglas-lum in MDCK cells. Co-staining of Daglas-lum with anti-TGN46 (Golgi marker; **D**), with BODIPY FL glibenclamide (endoplasmic reticulum marker; **E**), and with mitotracker orange (mitochondria marker; **F**). (**G**,**H**) Staining of Daglas-em (**G**) and Daglas-lum (**H**) with anti-GFP rabbit IgG antibody conjugated with Alexa Fluor 555 in semi-intact cells that were prepared by the treatment with streptolysin O. (**I**) Staining of Daglas-lum in Triton X-100-treated cells with anti-GFP rabbit IgG antibody conjugated with Alexa Fluor 555. All images were taken under a confocal laser scanning microscope. DIC, differential interference contrast.

FRET-based DAG probes for studying the cytosolic side of the plasma membrane (Daglas-pm) and endomembrane (Daglas-em) have been reported (Fig. 1B,C)^6^. These probes are advantageous because they can be directed to particular membrane surfaces of interest by inserting the appropriate membrane localization sequences (MLSs), thereby enabling the localized determination of DAG levels. To reveal DAG dynamics on the luminal side of endomembranes as well as the cytosolic side (Fig. 1A), we constructed a lumenal DAG probe using the p24 transmembrane domain, which mainly resides in the lumenal side of the Golgi apparatus and intermediate compartments, and functions as a cargo receptor of membrane vesicles that move from the endoplasmic reticulum to the Golgi apparatus^13^. The C1B domain from protein kinase Cβ (a specific DAG-binding domain) was introduced between a cyan fluorescent protein variant (Cerulean) and an enhanced yellow fluorescent protein variant (Venus); the domains were connected by rigid α-helical linkers comprising of repeated EAAAR sequences (Fig. 1B,C). We added a single di-glycine motif within one of the rigid linkers to act as a hinge. The C1B domain binds to DAG that is generated at cellular membranes, thereby inducing a substantial change in the conformation of the probe. This flip-flop-type conformational change results in increased FRET from Cerulean to Venus and allows for the stable observation of DAG by dual-emission ratio imaging. We named this probe ‘Daglas-lum’ (Fig. 1A,B).

First, the intracellular localization of Daglas-lum was addressed. When Daglas-lum was expressed in Madin-Darby canine kidney (MDCK) cells, fluorescence was observed in the Golgi apparatus and the endoplasmic reticulum, but not in mitochondria (Fig. 1D–F). Daglas-lum localization at the lumenal side of endomembranes was confirmed using semi-intact cells in which the plasma membrane was selectively permeabilized using the bacterial pore-forming toxin streptolysin O^14^. Under these conditions, cells expressing Daglas-em were labeled with anti-GFP antibodies (Fig. 1G), whereas cells expressing Daglas-lum were not labeled (Fig. 1H). In Triton X-100-treated MDCK cells, in which the endomembrane as well as plasma membrane were permeabilized, Daglas-lum was stained with anti-GFP antibody (Fig. 1I). Taken together, these data indicate that Daglas-lum localized to the lumenal side of endomembranes, including the Golgi apparatus and endoplasmic reticulum.

**Figure 2:**
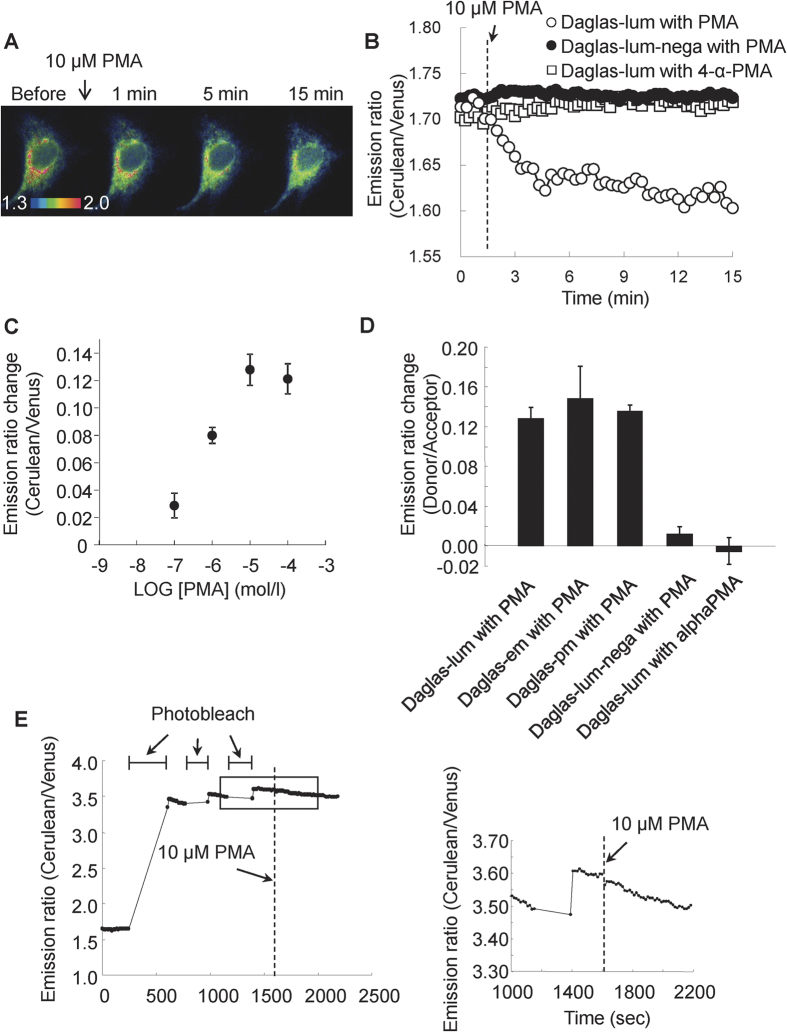
Imaging of FRET response of Daglas-lum. (**A**) Intensity modulated display (IMD) images of emission ratio change in Cerulean/Venus after 10 μM PMA stimulation of a MDCK cell expressing Daglas-lum. MDCK cells were transfected with a Daglas-lum vector 24 hours before imaging. (**B**) Time course of the Cerulean/Venus emission ratio of Daglas-lum after 10 μM PMA or 10 μM 4-α-PMA stimulation, and of Daglas-lum-nega after 10 μM PMA stimulation. (**C**) FRET change in response to various PMA concentrations. Kd was calculated as 0.35 ± 10^−7^ M by least squares method. (**D**) FRET responses of Daglas-lum, -pm and -em after 10 μM PMA stimulation in MDCK cells. The results are the means ± S.D. of emission ratio change from three different cells, after FRET response reached a plateau. (**E**) Photobleaching study of Daglas. Change in emission ratio after the MDCK cell expressed with Daglas-lum was excited at 540 ± 12.5 nm to photobleach the acceptor fluorophore, Venus. Subsequent stimulation with PMA of the MDCK cell did not affect emission ratio of Daglas-lum. The right panel shows an enlargement of the boxed area.

We next examined the responses of Daglas-lum to DAG. A single MDCK cell expressing Daglas-lum was excited at 440 ± 10.5 nm, and the ratio of the Cerulean emission (480 ± 15 nm) to the Venus emission (535 ± 13 nm) was monitored. The emission ratio of an MDCK cell expressing Daglas-lum is shown using intensity modulated display (IMD) images in Fig. 2A. After the addition of 10 μM phorbol 12-myristate 13-acetate (PMA), a DAG structural analogue that activates PKC isoforms by associating with their C1 domains, a distinct blue-shift was observed, demonstrating that a change in the Cerulean/Venus emission ratio of Daglas-lum had occurred. Figure 2B shows the time course of the Daglas-lum FRET signal. When cells were stimulated with PMA, a significant decrease in the Cerulean/Venus emission ratio was immediately observed. The emission ratio reached a plateau within 15 min. An inactive analog of PMA, 4-α-phorbol 12-myristate 13-acetate (4-α-PMA), did not significantly alter the Cerulean/Venus emission ratio of Daglas-lum (Fig. 2B). Additionally, the Daglas-lum-nega probe, the lipid-binding domain of which does not bind to DAG or other lipid messengers (12), was completely PMA insensitive (Fig. 2B). This decrease in the Cerulean/Venus emission ratio indicates that FRET from Cerulean to Venus increases when PMA binds to the C1B domain. The Daglas-lum FRET signal increased in a dose-dependent manner when PMA was added to the cells (Fig. 2C). The dissociation constant (Kd) was calculated as 0.35 ± 10^−7^ M. Daglas-pm, Daglas-em and Daglas-lum displayed very similar response to PMA at the plateau level (Fig. 2D). To further assess whether the response of Daglas-lum reflects FRET, photoinactivation of Venus within Daglas-lum by excitation at 540 ± 12.5 nm was carried out (Fig. 2E). Following photoinactivation, the emission ratio was increased due to the loss in energy transfer. The photobleached Daglas-lum did not significantly respond to PMA stimulation. This demonstrates that FRET actually increases in response to the DAG-dependent conformational change in Daglas. Taken together, these results indicate that the FRET between the Cerulean and Venus components of Daglas-lum is increased in response to PMA; thus, Daglas-lum enables the visualization of DAG on the lumenal side of the endomembrane.

**Figure 3:**
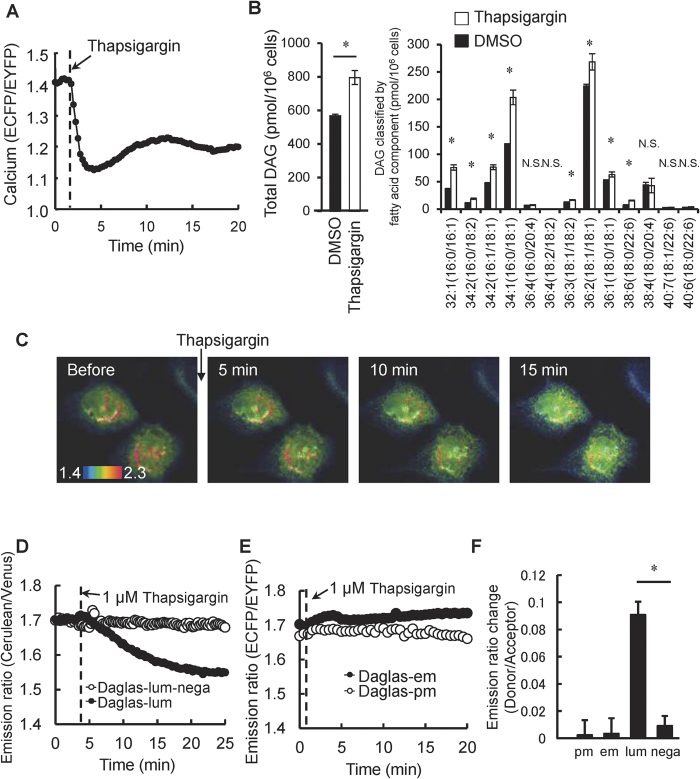
Thapsigargin-induced calcium increase induced lumenal DAG generation. (**A**) Time courses of emission ratio change (ECFP/EYFP) in YC2.1, a calcium probe, upon addition of 1 μM thapsigargin in MDCK cells. Emission ratio decrease indicates calcium increase. (**B**) Total DAG (Left panel) and fatty acid-classified DAG (Right panel) in MDMK cells were analyzed by LC/MS. *indicates statistical significant difference in DAG amount between DMSO and thapsigargin addition (p < 0.05). N.S. indicates no significant difference. Error bars indicate mean ± standard deviation (S.D.). (**C**) The images of emisstion ratio (Cerulean/Venus) after 1 μM thapsigargin addition to a MDCK cell. (**D**) Time course of emission ratio change (Cerulean/Venus) in Daglas-lum and Daglas-lum-nega upon addition of 1 μM thapsigargin. (**E**) Time courses of emission ratio change of Daglas-em and Daglas-pm upon addition of 1 μM thapsigargin. (**F**) Emission ratio changes at the point of 20 min of (**D**,**F**) were analyzed. Error bars indicate mean ± standard error (S.E.). *indicates statistical significant difference (p < 0.05).

Because calcium is reportedly implicated in DAG generation at the inner organelle membrane^8^, we investigated the effect of calcium on DAG dynamics. Calcium concentrations were increased using thapsigargin. When MDCK cells expressing the YC2.1 FRET-based calcium sensor were stimulated with 1 μM thapsigargin, the emission ratio decreased and then remained constant for more than 20 min, confirming that the calcium concentration had been increased. Next, the whole cell profile of DAG production was examined using mass spectrometry (LC-MS) (Fig. 3B). Major components including palmitoyl-oleoyl-diacylglycerol and dioleoyl-diacylglycerol (PODG and DODG, respectively) had increased in response to thapsigargin. These DAGs are produced in a sustained manner at endomembranes such as those of the Golgi apparatus and the endoplasmic reticulum^15^. In contrast, stearoyl-arachidonyl-diacylglycerol (SADG), which is temporarily generated from phosphatidylinositol 4,5-bisphosphates at the plasma membrane upon phospholipase C activation, was not generated. Next, the location of DAG production was investigated with Daglas-pm, Daglas-em and Daglas-lum. Daglas-pm did not respond (Fig. 3E,F). To our surprise, whilst Daglas-em also did not respond to thapsigargin (Fig. 3E,F), the Daglas-lum emission ratio decreased and reached a plateau 20 min later (Fig. 3C–F). These data suggest that thapsigargin-induced calcium elevation selectively resulted in lumenal DAG production under our experimental conditions.

**Figure 4:**
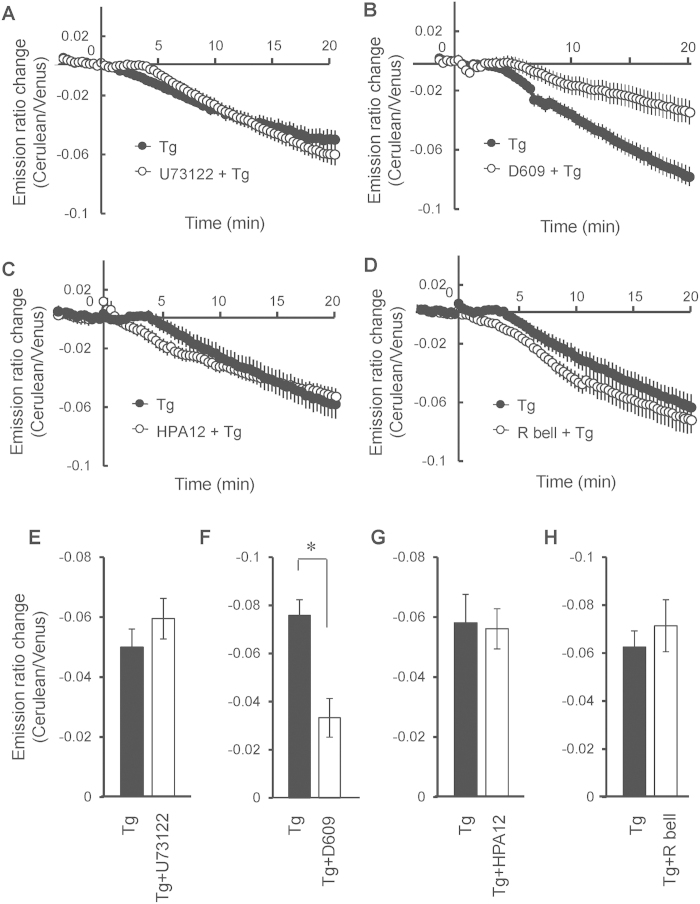
The effect of inhibitors on lumenal DAG evoked by calcium increase. (**A**) 1.33 μM U73122 was added 10 min before thapsigargin stimulation of MDCK cells. (**B**) 50 μg/ml D609 was treated 30 min before thapsigargin administration. (**C**) 2.5 μM HPA12 was added 10 min before thapsigargin administration. (**D**) 25 μM (R)-BEL was added 10 min before thapsigargin administration. (**E**–**H**) Emission ratio changes at the point of 20 min of (**A**–**D**) were analyzed. Error bars indicate mean ± standard error (S.E.). *indicates statistical significant difference (p < 0.001).

To investigate the mechanism underlying lumenal DAG production, several inhibitors were employed. U73122 was employed as an inhibitor of phosphatidylinositol-4,5-bisphosphate-specific phospholipase C^16^, which induces a transitory DAG increase at the plasma membrane. Pretreatment with U73122 for 10 min before thapsigargin stimulation did not change thapsigargin-induced lumenal DAG levels (Fig. 4A,E). D609 inhibits PC-PLC and sphingomyelin synthase (SMS), enzymes that have the potential to generate lumenal DAG^17^. Pretreatment with D609 10 min before the addition of thapsigargin inhibited thapsigargin-induced lumenal DAG production (Fig. 4B,F). HPA12 inhibits the ceramide transporter (CERT), which transports ceramide from the endoplasmic reticulum to the Golgi apparatus^18^. Ceramide is used as a substrate by SMS to produce DAG. (R)-Bromoenol lactone ((R)-BEL) inhibits adipocyte triacylglycerol lipase and phospholipase A2γ^19^. Thapsigargin-induced lumenal DAG production was unchanged after pre-incubation with either HPA12 or (R)-BEL for 10 min before thapsigargin stimulation (Fig. 4C,D,G,H). Taken together, these data suggest that calcium-induced lumenal DAG is regulated by D609-inhibited enzymes, such as SMS and PC-PLC.

## Discussion

The goal of this study was to develop a method to examine the real time dynamics of lipids in each leaflet of the lipid bilayer in living cell biomembranes. DAG-producing enzymes reside both in the cytosolic and in the lumenal sides of endomembranes. Therefore, we developed Daglas-lum which enables the visualization of DAG on the lumenal side of endomembranes in single living cells. Using Daglas-lum and Daglas-em, cytosolic and the lumenal DAG dynamics in MDCK cells were investigated. We revealed that thapsigargin-evoked calcium signaling was able to induce lumenal DAG at the endomembrane while the calcium elevation did not induce cytosolic DAG. Lumenal DAG production was abolished by an inhibitor of SMS and PC-PLC, that produces DAG at the lumenal side of endomembranes.

The extent of the transbilayer movement of DAG across the membrane remains controversial. DAG flip-flop is very fast based on the experimental results using DAG analogs in liposome vesicles. For example, a short-chain fluorescent analog and a long-chain sulfhydryl analog of DAG flip in less than one minute^20^,^21^. In contrast, our molecular dynamic simulation suggests that the lipid membrane environment affects the transbilayer movement of DAG^22^. For instance, 1-palmitoyl-2-oleoyl diacylglycerol (PODAG) flips 318.5 times during 80 μs in 1,2-di-arachidonoyl-*sn*-glycero-3-phosphocholine membranes. However, the flip-flop was not detected in 1-palmitoyl-2-oleoyl-*sn*-glycero-3-phosphocholine over an 80 μs calculation. In live MDCK cells, our previous results indicate that SM content in the plasma membrane regulates DAG flip-flop frequency^5^. In the present study, we found asymmetrical DAG production at the endomembrane in MDCK cells. The data suggests that DAG flip-flop rarely occurs at the endomembrane. Lumenal DAG may be implicated in cell signaling in the endoplasmic reticulum, such as endoplasmic reticulum stress mediated by unidentified DAG binding proteins.

The results obtained here demonstrate that thapsigargin does not trigger DAG production on the cytosolic side of the endomembrane. However, the Newton group demonstrated that thapsigargin-induced calcium elevation does leads to DAG generation at the cytosolic sides of the plasma membrane and the Golgi apparatus^8^. The differences in the cytosolic DAG production between our study and the Newton group study could be due to the studied cell type (MDCK vs. COS-7 cells). Alternatively, the differences might also be caused by the different thapsigargin concentration used, which was 1 μM in our study and 5 μM in the Newton group study^8^,^23^. We believe the observed difference is not due to the FRET probes because another DAG probe, Digda, also did not respond to 1 μM thapsigargin in MDCK cells (data not shown). Similar to Daglas-pm, Digda detects DAG at the cytoplasmic side of the plasma membrane. To support Newton’s results, we observed a positive signal from Digda when COS7 cells were treated with 5 μM thapsigargin (data not shown). Five micromolar is a very high thapsigargin working concentration. Kamiya *et al.* reported the protein content and cell viability of COS7 cells were not affected by thapsigargin treatment below 1 μM^24^. It has been reported that high thapsigargin concentrations induce loss of mitochondrial membrane potential, chromatin condensation and nucleus fragmentation^25^. We thus speculate that the observed differences may be due to the thapsigargin concentration used in the experiments.

Thapsigargin-induced lumenal DAG production was abolished by D609, an SMS and/or PC-PLC inhibitor^26^. Using freeze-fracture immune-electron microscopy, we recently demonstrated that in the plasma membrane, 82.6% of PC and 87.9% of SM are distributed in the outer leaflet of the lipid bilayer, suggesting that these lipids are enriched in the exoplasmic leaflet of the organelle membrane. SM is synthesized in the exoplasmic leaflet of the Golgi apparatus and the plasma membrane^27^,^28^. Consistent with this observation, the putative active sites in SMS1 and SMS2 are positioned on the exoplasmic leaflet^11^. The distribution of PC-PLC at the ectoplasmic membrane surface in human NK cells has also been reported^29^. Our results indicate that cellular calcium homeostasis regulates lipid metabolism inside organelles.

## Materials and Methods

### Materials

Phorbol 12-myristate 13-acetate (PMA) and 4-α-phorbol 12-myristate 13-acetate (4-α-PMA) were purchased from the Sigma Chemical Co. (St. Louis, MO). Hank’s balanced salt solution (HBSS) and Dulbecco’s modified Eagle’s medium (DMEM) were obtained from Invitrogen (Carlsbad, CA). Alexa Fluor® 555 conjugated anti-green fluorescent protein rabbit IgG fraction, Mitotracker Orange CMTMRos and ER-Tracker™ Red (BODIPY® FL glibenclamide) were obtained from Invitrogen Molecular Probes Inc. (Eugene, OR). All other chemicals were of analytical reagent grade.

### Plasmid construction

To construct Daglas-lum cDNA, cerulean cDNAs fragments with a signal sequence^30^, C1B with Ln1 and Ln2, Venus with Ln3, and MLS3 were generated by standard PCR. Daglas-pm and Daglas-em were generated as previously described previously^6^. Each cDNA fragment was subcloned into a pCR-Blunt II-TOPO vector (Invitrogen Co., Carlsbad, CA). All of the cloning enzymes were from TOYOBO (Osaka, Japan) and were used according to the manufacturer’s instructions. All of the PCR fragments were sequenced with a 3100 genetic analyzer (Applied Biosystems, CA). cDNA encoding Daglas-lum was subcloned into the *Hin*dIII and *Xha*I sites of the mammalian pcDNA3.1(+) expression vector (Invitrogen Co., Carlsbad, CA).

### Cell culture and transfection

MDCK cells were cultured in DMEM supplemented with 10% fetal calf serum at 37 °C in 5% CO_2_. Cells were plated onto glass bottom dishes, transfected with the LipofectAMINE 2000 reagent, and left for 24 h at 37 °C in 5% CO_2_.

### Imaging of cellular diacylglycerol and intracellular calcium

The culture medium was replaced with HBSS for fluorescent imaging. The cells were imaged at 25 °C on a Carl Zeiss Axiovert 200 microscope with a cooled CCD camera CoolSNAP HQ (Roper Scientific Inc. Tucson, AZ) controlled by the MetaFluor software (Universal Imaging, West Chester, PA). The exposure time at 440 ± 10.5 nm excitation was 100 ms and 300 ms for Venus and Cerulean, respectively. Fluorescence images were obtained through 480 ± 15 nm and 535 ± 13 nm filters with a 40x oil immersion objective (Carl Zeiss, Jena, Germany). The images in this study were displayed in intensity modulated display (IMD) mode, in which the intensity of the fluorescence is considered to make the images displayed with Cerulean/Venus ratio images.

### Cell staining

All manipulations were performed at room temperature. MDCK cells grown on coverslips were washed with phosphate buffered saline (PBS) and then fixed for 20 min with 3% paraformaldehyde in PBS. The cells were washed with PBS and quenched with 50 mM NH_4_Cl for 10 min. After washing with PBS, cells were permeabilized by treatment with 0.2% Triton X-100 for 5 min. The specimens were blocked with 0.2% gelatin in PBS for 60 min. After 30 min treatment with the first anti-Golgi antibody, cells were washed and labeled with the fluorescent second antibody. The stained cells were washed and mounted in Mowiol (Sigma-Aldrich). To stain the mitochondria and endoplasmic reticulum, MDCK cells were incubated for 10 min at 25 °C with 1 μM Mitotracker and 1 μM endoplasmic reticulum-Tracker Red, respectively. After cells were washed once with HBSS, the samples were observed under a Zeiss LSM 510 confocal microscope equipped with a C-Apochromat 63XW Korr (1.2 n.a.) objective.

### Preparation of semi-intact cells

Semi-intact cells were prepared according to the previously reported procedure^14^ with slight modification. Briefly, MDCK cells expressing Daglas-lum and -em were cooled to 4 °C, washed with PBS three times and incubated with streptolysin O (SLO) for 4 min on ice. Excess SLO was removed by washing three times with PBS and then the cells were warmed to 33 °C for 2 min to allow pore formation in the presence of the buffer (20 mM HEPES-KOH, pH 7.3, 110 mM CH_3_COOK, 5 mM CH_3_COONa, 2 mM (CH_3_COO)_2_Mg and 1 mM EGTA). The permeabilized cells were incubated with Alexa Fluor 555-conjugated anti-GFP antibody for 60 min at 25 °C and observed under a Zeiss LSM 510 confocal microscope.

### Mass spectrometry analysis of DAG

To detect DAG by mass spectrometry, liquid chromatography-electrospray ionization mass spectrometry (LC-MS) (Ultimate 3000 LC system, Thermo-Fisher Scientific, Waltham, MA, USA) was used. All lipids from the MDCK cells were extracted using a butanol solution that is for the hydrophilic lipids, and a hexane/ethyl acetate solution for the relatively neutral lipids. Both solutions containing DAG were mixed, and applied to an Acclaim PepMap100 C18 (3 μm, 150 × 1.0 mm i.d.) column (Thermo-Fisher Scientific). Separated lipids were ionized by an electrospray ionization (HESI II probe) source, and then applied to a TSQ Vantage mass spectrometer (Thermo-Fisher Scientific). See the reference for detailed information^31^.

## Additional Information

**How to cite this article**: Ueda, Y. *et al.* Asymmetrical diacylglycerol dynamics on the cytosolic and lumenal sides of a single endomembrane in living cells. *Sci. Rep.* **5**, 12960; doi: 10.1038/srep12960 (2015).
